# Development of a near infrared novel bioimaging agent *via* co-oligomerization of Congo red with aniline and *o*-phenylenediamine: experimental and theoretical studies†

**DOI:** 10.1039/c9ra05814a

**Published:** 2019-11-13

**Authors:** Neetika Singh, Prabhat Kumar, Raj Kumar, Elham S. Aazam, Ufana Riaz

**Affiliations:** Materials Research Laboratory, Department of Chemistry, Jamia Millia Islamia New Delhi 110025 India ufana2002@yahoo.co.in; Advanced Instrumentation Research Facility, Jawaharlal Nehru University NewDelhi 110067 India; School of Life Sciences, Jawaharlal Nehru University New Delhi 110067 India; Chemistry Department, Faculty of Science, King Abdul Aziz University Jeddah 23622 Saudia Arabia

## Abstract

With a view to study the effect of insertion of a multifunctional dye moiety on the photo physical properties of conducting polymers, the present paper reports for the first time the homopolymerization and co-oligomerization of Congo red (CR) dye with aniline and *o*-phenylenediamine. The co-oligomerization was established by Fourier transform infrared spectroscopy (FTIR), nuclear magnetic resonance spectroscopy (^1^H-NMR), and ultraviolet-visible (UV-vis) spectroscopy while the morphology was examined using X-ray diffraction (XRD) and scanning electron microscopy (SEM) techniques. The theoretical as well as experimental data of ^1^H-NMR as well as IR studies confirmed the co-oligomer formation while ultraviolet-visible spectroscopy studies revealed a dynamic change in the optical properties upon variation of co-oligomer composition. X-ray diffraction studies established a crystalline morphology of oligomers. Live cell confocal imaging studies revealed that the co-oligomers could be effectively used in NIR imaging.

## Abstract 2

To study the effect of insertion of a multifunctional dye moiety on the photophysical properties of conducting polymers, the present paper reports for the first time the homopolymerization and co-oligomerization of Congo red (CR) dye with aniline and *o*-phenylenediamine.

## Graphical abstract

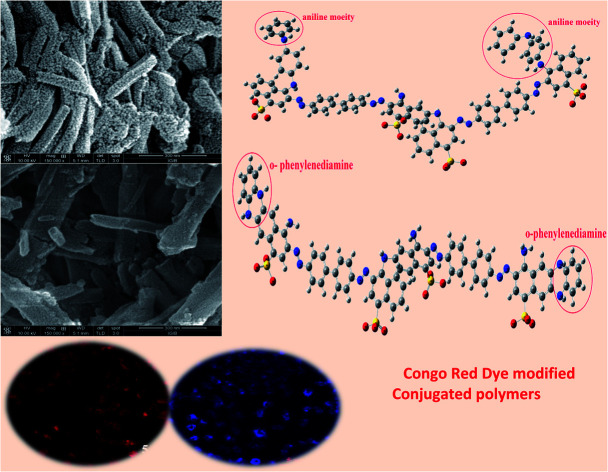

## Introduction

The spectral and photo-physical properties of conjugated polymers such as polyaniline (PANI),^1,2^ poly (*o*-phenylenediamine) (POPD),^3,4^ polypyrrole (Ppy),^5,6^ polythiophene (PTh),^7,8^ polyethylene-dioxythiophene (PEDOT)^9,10^*etc.* are highly tunable in nature and can be altered by merely substituting the monomers with donor/acceptor moieties to obtain emission in the near infrared (NIR) region. These conjugated polymers find promising applications in solar cells,^11^ biosensing^12,13^ and bioimaging.^14–16^ Several investigations have been reported on the design of donor–acceptor based conjugated polymers for their potential application in bio-imaging and photodynamic therapy.^17–20^ However, scant literature is available on the design of conjugated polymers through the insertion of azo dyes in the main chain *via* co-oligomerization/doping.^21,22^ Azo compounds are extensively used in optical storage,^23^ optical switching,^24^ non-linear optical devices^25^ as well as various kinds of photonic devices.^26^ Azo dyes have specific physico-chemical and biological properties which are widely utilized in cellullar staining to visualize cellular components and metabolic processes.^27–30^ With the aim to study the influence of the multifunctional dye moiety on the structural properties of conventional conjugated polymers, the present work reports the chemical oxidative co-oligomerization of Congo red with aniline and *o*-phenylenediamine. The main aim of this investigation was to prepare a water soluble non-cytotoxic NIR emitting polymer which could be utilized as a bioimaging agent. The synthesized co-oligomers were characterized using Fourier transform infrared spectroscopy (FTIR), nuclear magnetic resonance spectroscopy (^1^H-NMR), ultraviolet-visible (UV-vis) as well as fluorescence spectroscopy while the morphology was investigated using X-ray diffraction (XRD) and scanning electron microscopy (SEM) studies. The cell viability was examined using the methyl tetrazolium (MTT) assay and the imaging capability of the polymers was studied *via* live cell imaging of human cervical tumor (HeLa) cells.

## Experimental

### Materials and methods

Congo red (SD Fine limited), Aniline (Fisher Scientific), *o*-phenylenediamine (Sigma Aldrich, USA), ferric chloride (Merck, India), ethanol (Merck, India), hydrochloric acid (0.5 N HCl) (Merck, India), *N*,*N*-dimethyl pyrrolidone (Merck, India) were used without further purification.

Congo red dye was purified by a reported method.^31^ Approximately 20 g of the dye was dissolve distilled water (100 ml) and filtered. The dye solution was then heated to boiling on a heating mantle and sodium acetate was added in excess to precipitate the dye. The obtained dye precipitate was then filtered on a Buchner funnel and boiled in ethanol (150 ml). The suspended dye was then removed from alcohol by filtration. The digestion with alcohol was repeated several times till a small amount of dye was dissolved by the alcohol.

### Polymerization of Congo red dye

Congo red (CR) (1 g, 0. 0014 mol) was added to 250 ml 3-necked round bottom flask containing in deionized water (50 ml) and 0.5 M HCl (100 ml). Ferric chloride (1 g, 0.006 mol) dissolved in water (20 ml) was added to the above reaction mixture drop by drop with the help of a burette. The reaction mixture was stirred on a magnetic stirrer equipped with thermometer and N_2_ gas for 24 h at room temperature. The obtained polymer was then centrifuged and dried in a vacuum oven for 72 h at 70 °C. The purification was carried out *via* re-precipitation technique by suspending the obtained oligomer in 250 ml *N*-methyl pyrrolidone (NMP). The suspension was occasionally stirred and after 30 days, the insoluble part was separated by filtration. The insoluble part was then well rinsed with methanol and dried at ambient atmosphere. The solutions containing the soluble part were added drop-wise into 1.5 L of methanol containing 15 mL of concentrated sulphuric acid. The precipitate was collected on a filter paper and dried at room temperature. The oligomer was designated as PCR and the yield obtained was 78%.

### Co-polymerization of CR dye using aniline and *o*-phenylenediamine

Congo red (CR) (1.00 g, 1.4 × 10^−3^ mol) and aniline (ANI) (0.15 ml, 1.6 × 10^−3^ mol) were added to 100 ml Erlenmeyer flask containing water (20 ml). Around 100 ml of 0.5 M HCl was also added in the above solution. Ferric chloride (1 g, 6.0 × 10^−3^ mol) dissolved in 20 ml distilled water was added to the reaction mixture which was subjected to stirring on a magnetic stirrer equipped with thermometer and nitrogen gas for 24 h at room temperature. Similar procedure was adopted for the synthesis of co-oligomers of Congo red (CR) (1.00 g, 1.4 × 10^−3^ mol) and *o*-phenylenediamine (OPD) (0.145 g, 1.39 × 10^−3^ mol). The synthesized co-oligomers were centrifuged and dried in a vacuum oven for 72 h at 70 °C to ensure complete removal of water and impurities. The purification of the synthesized co-oligomers was carried out in a similar way mentioned for PCR. The synthesized co-oligomers were designated as: PCR-*co*-PANI, and PCR-*co*-POPD and the yields were calculated to be 72% and 75% respectively.

### Characterization

IR spectra of co-oligomers were taken on FT-IR spectrophotometer (Shimadzu, Model IRA Affinity-1) in the form of KBr pellets. The integrated absorption coefficient 
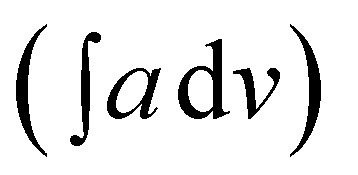
 was determined using the IRA Affinity-1 software through Gaussian Lorentzian curve fittings. Ultraviolet-visible light (UV-vis) spectra were taken on UV-vis spectrophotometer (Shimadzu, Model UV-1800). The viscosity of the co-oligomers was determined at 25 °C temperature using Ubbehlode viscometer. XRD patterns of the co-oligomers were recorded on a powder diffractometer (Philips, Model PW 3710) (using a nickel-filtered Cu-Kα radiation). Peak parameters were analyzed *via* Origin Pro 8 software. Fluorescence studies were performed on fluorescence spectrophotometer Fluorolog @ 3–11 (Horiba) The quantum yield was calculated as per the method reported in our earlier studies.^4^

### Gaussian calculations

The calculations for geometry optimization, ^1^H-NMR and vibrational spectra were performed *via* Gaussian 09 W software package.^32^ The geometries were fully optimized at the B3LYP level using 6-31G (d,p) basis set. The vibrational frequencies were computed using the same basis set. The UV spectra were of optimized geometric structures were simulated at TD-DFT/B3LYP using 6-31G (d,p) basis set, while the ^1^H-NMR spectra were computed using the gauge independent atomic orbital (GIAO) method.

### Cell culture and MTT assay studies

Human cervical cancer cell (HeLa) was procured from National Centre for Cell Science, Department of Biotechnology, Pune, India. The live cell imaging studies were performed using live cell microscope (Ti, Nikon, TOKYO, JAPAN) at 10× magnification as per method reported in our previous studies. MTT assay was used for the evaluation of cell viability as per method reported in our previous studies.^3,4^ 96-well plate was used to seed cells having density of 5 × 10^3^ cells per well and kept overnight for attachment. Media was replaced and cells were exposed for 24 h to different concentrations of the oligomers and 20 μl containing 5 mg ml^−1^ of MTT was poured to each well 4 h before the incubation would complete. The media was replaced and 200 μl of DMSO was added followed by incubation at room temperature for another 10 min. ELISA reader was used to observed absorbance at 595 nm. The % viability was calculated using formula reported in previous studies.^3,4^
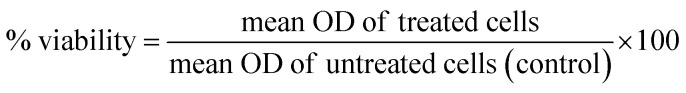


## Results and discussion

### Viscosity average molecular weight of PCR and its co-oligomers

**Scheme 1:**
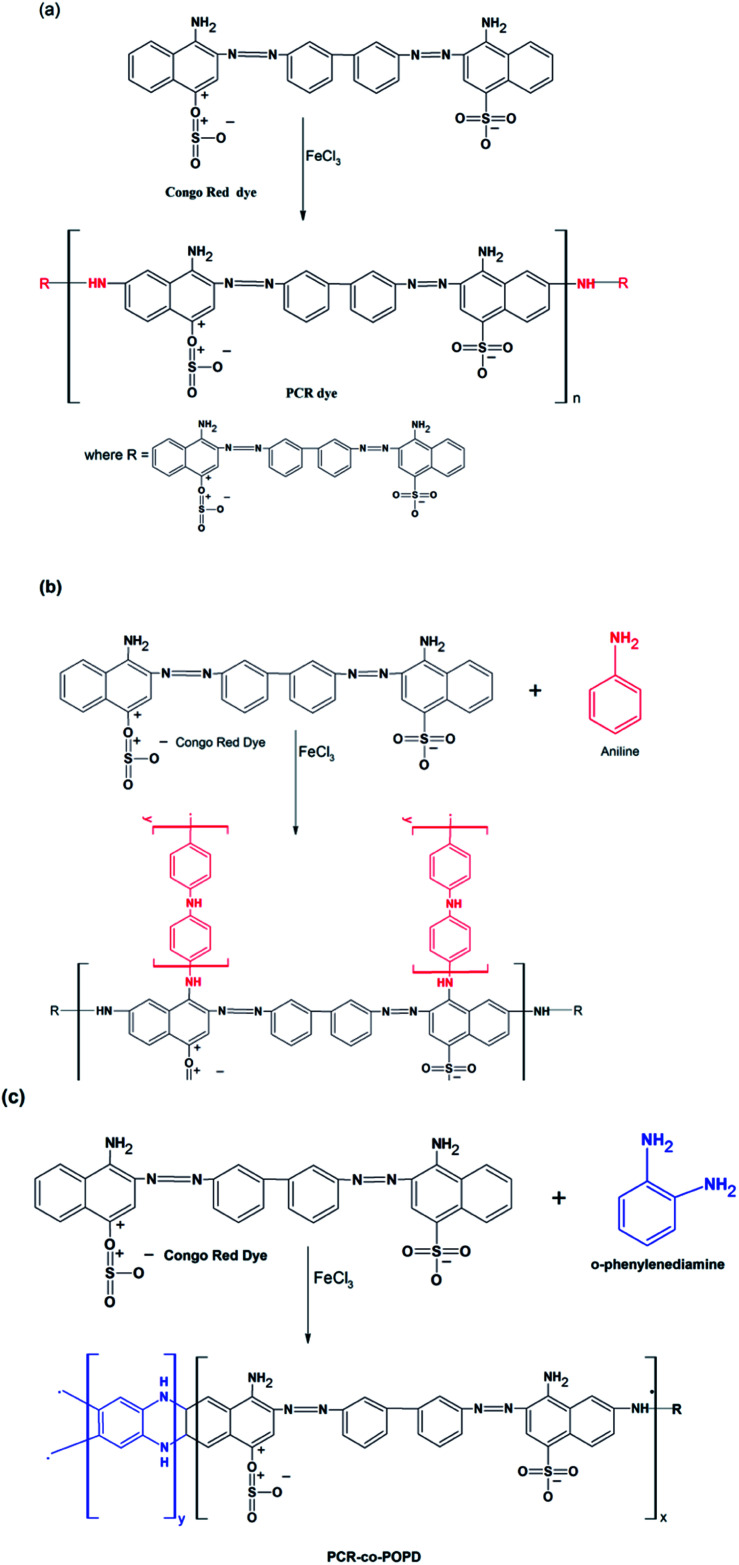
Chemical structures of (a) PCR, (b) PCR-*co*-PANI, (c) PCR-*co*-POPD.

The chemical structures of PCR, PCR-*co*-POPD and PCR-*co*-PANI are shown in Scheme 1(a–c). The predicted oligomeric structures were further established by comparing the experimental and theoretical, IR, ^1^H-NMR as well as UV data which is discussed in the later sections. The intrinsic viscosity [*η*] values of PCR, PCR-*co*-PANI and PCR-*co*-POPD in NMP were calculated to be 0.11, 0.13, and 0.15 respectively. The viscosity average molecular weight was determined by employing the Mark Houwink equation as per method reported in our earlier studies.^33^ The average molecular weight was calculated to be 5117 for PCR, 6179 for PCR-*co*-PANI and 6814 for PCR-*co*-POPD respectively (given in ESI as Table S1†). PCR-*co*-POPD showed highest intrinsic viscosity due to its rigid chain structure as both the amino groups were linked to the aromatic ring of PCR molecule while in case of PCR-*co*-PANI a single NH linkage was present that comparably imparted flexibility to the oligomeric structure. The co-oligomerization of PCR was further confirmed by the experimental as well as theoretical IR and ^1^H-NMR spectra.

### Calculation of optimized geometry, frontier molecular orbitals and band gap values

**Fig. 1:**
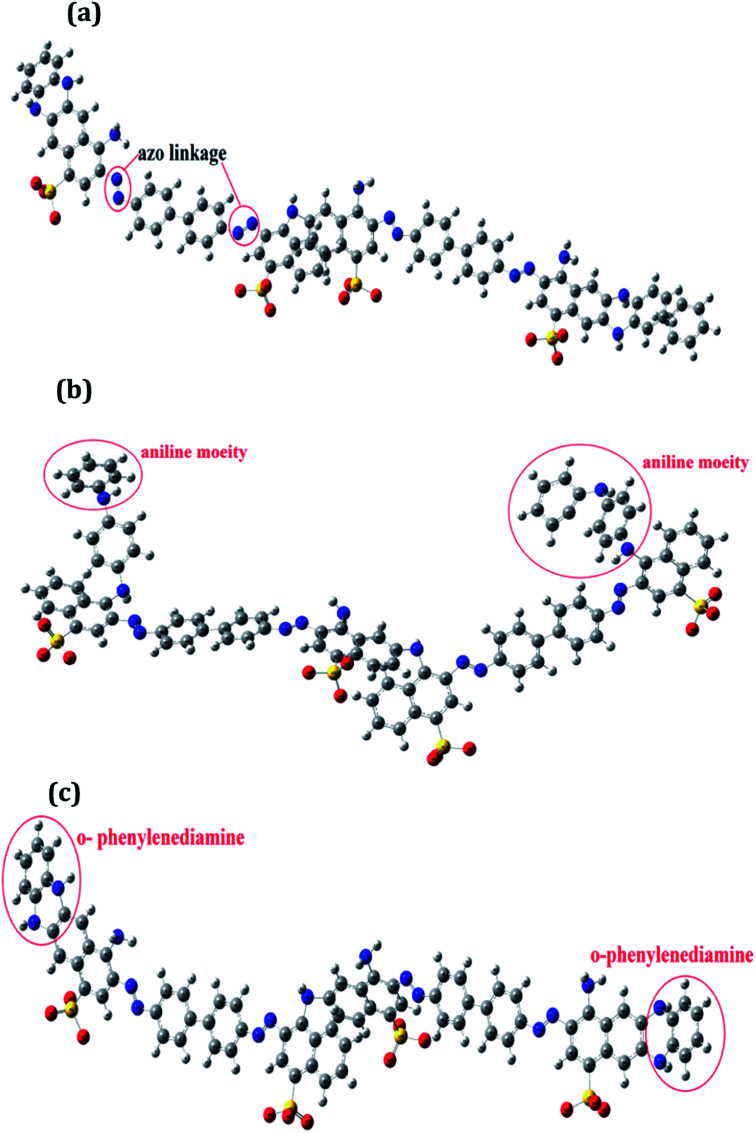
Optimized geometries of (a) PCR, (b) PCR-*co*-PANI, (c) PCR-*co*-POPD.

The optimized geometrical structures for the lowest conformers obtained are represented in Fig. 1(a–c). The structures of PCR, PCR-*co*-PANI and PCR-*co*-POPD were optimized using dimers of CR, Aniline and *o*-phenylenediamine. The dimer of PCR revealed a planar configuration, Fig. 1(a), while PCR-*co*-PANI, Fig. 1(b), showed twisted configuration particularly around the NH linkages between PANI and PCR dimers. However, in case of PCR-*co*-POPD, Fig. 1(c), the presence of two NH linkages between the aromatic rings of PCR and POPD inhibited the twisting of the oligomeric chain and maintained the planar configuration of PCR. For PCR, the C–C bond length was computed to be 1.47 Å while the C=C bond length was found to be 1.42 Å. The N=N bond length was observed to be 1.29 Å while the C–N bond length was found to be 1.35 Å. In case of PCR-*co*-PANI, the C–C, C–N, N=N and N–H bond lengths were found to be 1.40 Å, 1.46 Å, 1.24 Å and 2.03 Å. For PCR-*co*-POPD, the C–C, C–N, N=N and N–H bond lengths were computed to be 1.40 Å, 1.46 Å, 1.23 Å and 1.12 Å respectively.

**Fig. 2:**
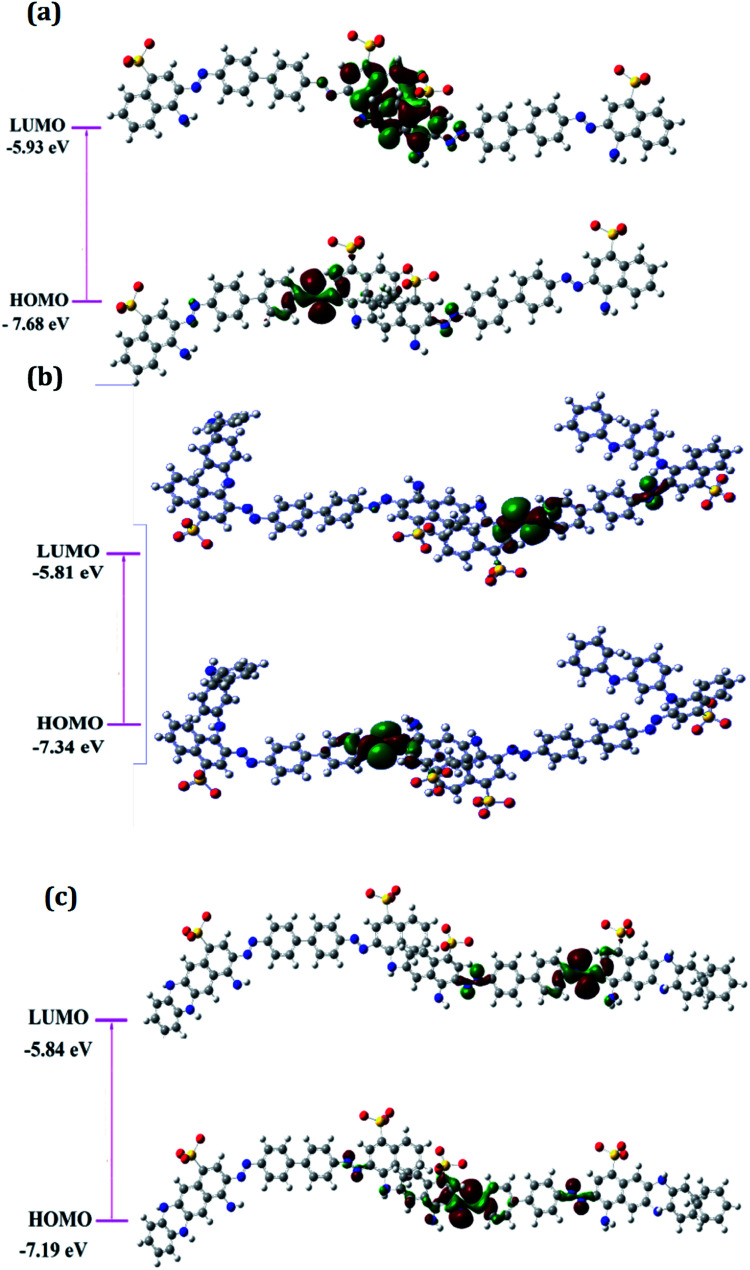
Frontier molecular orbital distributions in (a) PCR, (b) PCR-*co*-PANI (c) PCR-*co*-POPD.

The distribution of frontier molecular orbitals is depicted in Fig. 2(a–c). The HOMO orbitals for all the oligomers were noticed to be located on the aromatic rings bearing the N=N linkage and the computed HOMO values were observed to be highest for PCR and lowest for PCR-*co*-POPD. The HOMO levels were found to be significantly influenced upon insertion of aniline and *o*-phenylenediamine moieties. The electron density distributions at LUMO orbitals were noticed to be localized around the aromatic ring bearing the sulphonic acid groups. The computed values of HOMO were found to be −7.68 eV for PCR, −7.34 eV for PCR-*co*-PANI and −7.19 eV for PCR-*co*-POPD. The band gap was calculated to be 1.75 eV for PCR, 1.53 eV for PCR-*co*-PANI and 1.35 eV for PCR-*co*-POPD. The band gap values were noticed to be significantly low upon insertion of *o*-phenylene diamine.

### Confirmation of co-oligomerization of PCR *via*^1^H-NMR and IR studies

The ^1^H-NMR spectrum of PCR (given in ESI as Fig. S1(a)†), revealed a peak at *δ* = 4.5 ppm confirming the dimerization of PCR *via* N–H linkage, while the peak at *δ* = 7.64 ppm were attributed to the protons of the ring bearing the NH_2_ group, Table 1. The aromatic ring proton between the N=N and SO_3_-functional groups was noticed at *δ* = 8.27 ppm while the protons associated with the aromatic ring fused to amino benzene sulphonic acid were seen at *δ* = 7.48 ppm, 8.42 ppm and 8.71 ppm. The protons of the aromatic ring adjacent to the N=N linkage were found at *δ* = 8.07 ppm and 8.09 ppm. The theoretical spectrum of PCR (given in ESI as Fig. S1(a)† as inset) revealed peaks similar to the ones obtained from the experimental data and therefore confirmed the oligomerization of PCR. The experimental ^1^H-NMR spectrum of PCR-*co*-PANI (given in ESI as Fig. S1(b)†), exhibited a prominent peak associated with the presence of NH linkage of PANI at *δ* = 5.8 ppm while the theoretical spectrum revealed the same peak at *δ* = 5.6 ppm. The peaks between *δ* = 7–7.3 ppm were correlated to aromatic ring protons of PANI and PCR which were noticed around *δ* = 6.5–7.5 ppm in the theoretical spectrum. The proton of the NH_2_ attached to the aromatic ring was noticed at *δ* = 7.55 ppm in the experimental spectrum while it was found at *δ* = 7.58 ppm in the theoretical spectrum. The aromatic protons of aniline ring were observed at *δ* = 7.02 ppm, 7.15 ppm and 7.28 ppm while the protons of the aromatic ring fused with the amino benzene sulphonic acid ring were noticed at *δ* = 8.69 ppm, 8.40 ppm and 7.31 ppm. The peaks were found to be in close agreement with the theoretical spectrum. Similarly, the ^1^H-NMR spectrum of PCR-*co*-POPD revealed a pronounced peak at *δ* = 5.6 ppm associated with –NH proton of POPD while the peak of the NH proton of PCR was observed at *δ* = 4.4 ppm. The protons of POPD ring appeared at *δ* = 7. 03 ppm, 7.15 ppm and 7.19 ppm while the theoretical spectrum showed the same protons at *δ* = 7.1 ppm and 7.2 ppm. The molar ratios of the co-oligomer composition were calculated by comparing the integrated areas of –NH protons as reported in our previous studies.^3,32^ The integrated areas of NH protons were calculated to be 24 : 76 (PCR : PANI) for PCR-*co*-PANI while it was observed to be 42 : 58 (PCR : POPD) for PCR-*co*-POPD. The co-oligomer composition was observed to be almost equal to the feed molar ratio in case of PCR-*co*-POPD while it was noticed to be different than the feed ratio for PCR-*co*-PANI. This could be attributed to higher reactivity ratio of aniline monomer and its ability to undergo homopolymerization forming a block co-oligomer. The results thus confirmed the co-oligomerization of Congo red with aniline and *o*-phenylenediamine. As the theoretical spectrum was found to be in close agreement with the experimental spectrum in all the three cases, the structures of the oligomers were confirmed to be similar to the proposed structures as depicted in Scheme 1(a–c).

**Table 1:** Experimental and theoretical ^1^H-NMR data of PCR, PCR-*co*-PANI and PCR-*co*-POPD

Polymer/co-oligomer	Atom (labels shown in figure)	Chemical shift (ppm) (exp.)	Chemical shift (ppm) (theoretical)
PCR	N–H (h)	4.50	4.51
	Aromatic NH_2_ (f)	7.64	7.63
	Protons of aromatic ring adjacent to amino benzene sulphonic acid ring (a, b, g)	8.71	8.91
		8.42	8.40
		7.48	8.31
	Aromatic ring proton between N=N and SO_3_^−^ group (c)	8.27	8.21
	Aromatic ring protons on right side of N=N linkage (d and e)	8.09	8.10
		8.07	8.11
PCR-*co*-PANI	N–H (h)	4.7	5.5
	N–H of PANI (i)	5.8	5.6
	Aromatic NH_2_ (f)	7.55	7.58
	Protons of aromatic ring of PANI (j–l)	7.02	7.11
		7.15	7.24
		7.28	7.33
	Protons of aromatic ring adjacent to amino benzene sulphonic acid ring (a, b, g)	8.69	8.55
		8.40	8.46
		7.31	7.27
	Aromatic ring proton between N=N and SO_3_^−^ group (c)	8.25	8.33
	Aromatic ring protons on right side of N=N linkage (d and e)	8.06	8.11
		7.90	8.13
PCR-*co*-POPD	N–H (h)	4.4	4.5
	N–H of POPD (i)	5.6	5.5, 5.7
	Aromatic NH_2_ (f)	7.77	7.8
	Protons of aromatic ring of POPD (j–l)	7. 03	7.1
		7.15	7.2
		7.19	—
	Protons of aromatic ring adjacent to amino benzene sulphonic acid ring (a, b, g)	8.66	8.9
		8.40	8.7
		7.43	7.4
	Aromatic ring proton between N=N and SO_3_^−^ group (c)	8.24	8.3
	Aromatic ring protons on right side of N=N linkage (d and e)	8.03	8.1
		7.94	7.9

The IR data of PCR and its co-oligomers is given in Table 2 (Fig. S2(a–c) provided in ESI†). The homopolymer PCR (given in ESI as Fig. S2(a)†) revealed a broad and diffuse N–H stretching vibration region with small humps at 3382 cm^−1^, 3263, cm^−1^, 3155 cm^−1^ and 3112 cm^−1^. The theoretical IR spectrum also revealed peaks in the same region and the 
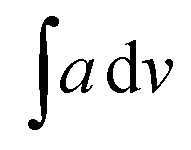
 value of NH region in the experimental spectrum was computed to be 357.8. The imine stretching peak was noticed at 1640 cm^−1^ while the peaks associated with quinonoid rings were found at 1566 cm^−1^ and 1517 cm^−1^ respectively.

**Table 2:** FTIR data of PCR, PCR-*co*-PANI and PCR-*co*-POPD

Polymer/co-oligomer	Functional group	Peak position/cm^−1^ (experimental)	Peak position/cm^−1^ (theoretical)
PCR	N–H stretching	3382, 3263, 3155, 3112	3482, 3384, 3366, 3348
	Imine stretching	1640	1638
	C=C stretching (quinonoid)	1566, 1517	1562, 1512
	C=C stretching (benzenoid)	1452, 1388, 1380	1450, 1388, 1386
	C–N stretching	1242	1242
	C–C stretching (benzenoid)	1354, 1323	1350, 1332
	C–H bending	1164, 1070	1170, 1080
	Substituted phenyl ring	956, 852, 757, 732, 709	954, 858, 756, 720, 702
PCR-*co*-PANI	N–H stretching	3328, 3280, 3161, 3014	3348, 3276, 3150, 3012
	C–H stretching	2974, 2869	2916, 2880
	Imine stretching	1677, 1627	1674, 1620
	C=C stretching (quinonoid)	1521	1530
	C=C stretching (benzenoid)	1442, 1406, 1384	1440, 1404, 1386
	C–N stretching	1236	1242
	C–H bending	1062	1062
	Substituted phenyl ring and phenazine skeleton	948, 850, 796, 732	936, 846, 792, 738
PCR-*co*-POPD	N–H stretching	3294, 3263	3298, 3262
	C–H stretching	2964, 2929, 2863	2960, 2989, 2860
	Imine stretching	1716	1714
	C=C stretching (quinonoid)	1568, 1450	1561, 1453
	C–C stretching (benzenoid)	1388, 1321	1381, 1327
	C–N stretching	1240	1246
	C–H bending	1070	1075
	Substituted phenyl ring, phenazine skeleton	958, 891, 852, 806, 734	958, 895, 850, 805, 733

The vibrations of the benzenoid ring appeared at 1452 cm^−1^, 1388 cm^−1^, 1380 cm^−1^, respectively. The CN stretching vibration peak was observed at 1242 cm^−1^. The peaks associated with substituted benzene ring and phenazine skeleton were observed around 956–709 cm^−1^.

The IR data of PCR-*co*-PANI, Table 2 (Given in ESI as Fig. S2(b)†), also revealed broad NH stretching vibration spanning between 3328-3014 cm^−1^ with the 
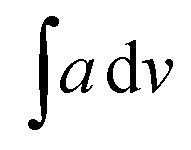
 value of 448.6. The area of the NH region was noticed to be higher than pure PCR. The imine peaks were detected at 1677 and 1627 cm^−1^. The peaks correlated to quinonoid and benzenoid ring stretching vibrations were noticed at 1521 cm^−1^, 1442 cm^−1^, 1406 cm^− 1^ and 1384 cm^−1^ respectively. The CN stretching vibration peak was found at 1236 cm^−1^ while the region spanning between 948–732 cm^*−*1^ corresponded to the vibrations of the aromatic rings of PCR and PANI. The theoretically computed spectrum was found to be in close agreement with the experimental spectrum. Similarly, the IR spectrum of PCR-*co*-POPD (given in ESI as Fig. S2(c)†), exhibited NH stretching vibration band between 3294–3263 cm^−1^ with the 
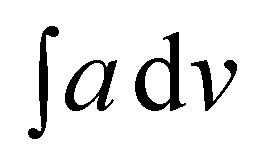
 value of 535.9 while the peaks associated with quinonoid and benzenoid ring stretching vibrations appeared at 1568 cm^−1^, 1450 cm^−1^, 1388 cm^−1^ and 1321 cm^−1^ respectively.^32,33^ The CN stretching vibration peak was found at 1240 cm^−1^. The peaks associated with the phenazine skeleton and di-substituted benzene ring were observed in the range of 958–734 cm^*−*1^. The theoretical IR spectra of the co-oligomers of PCR were found to be matching with the experimental spectra and thus confirmed the structure of the hompolymers and co-oligomers as shown in Scheme 1(a–c).

### Morphological characteristics of the co-oligomers analyzed *via* XRD and SEM studies

**Fig. 3:**
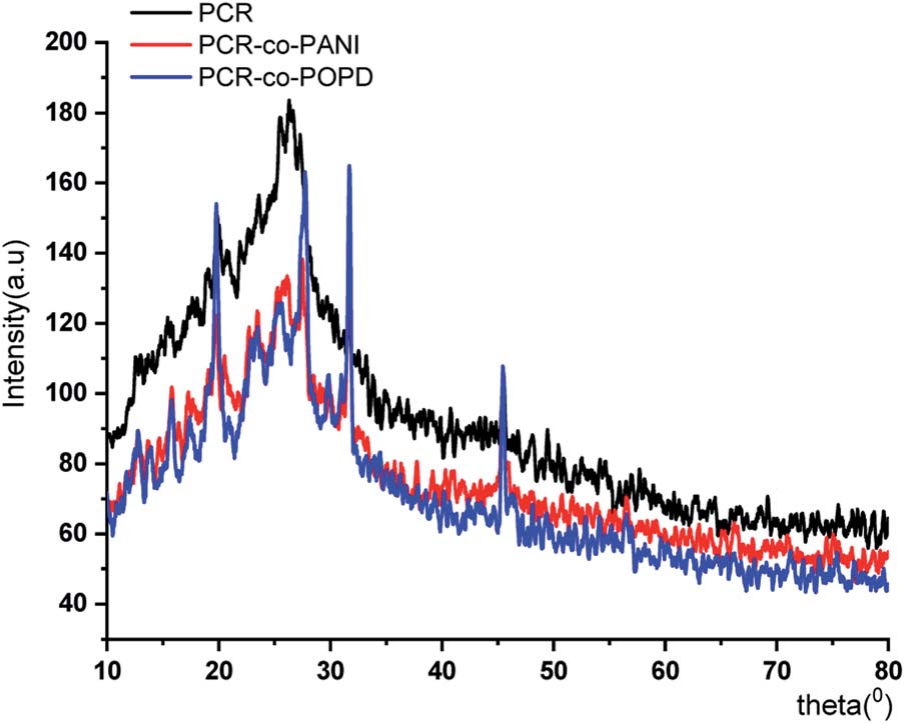
XRD of PCR and its co-oligomers.

The XRD pattern of PCR, Fig. 3, showed sharp peaks at 2*θ* = 27.25°, 32.18°, 37.84°, 44.91° and 45.33° corresponding to (101), (111), (117), (124) and (134) planes respectively.^3,32,33^ The XRD pattern of PCR-*co*-PANI revealed a pronounced high angle peak at 2*θ* = 31.7° indicating amorphous structure of the co-oligomer. The XRD profile of PCR-*co*-POPD exhibited three intense peaks at 2*θ* = 19.58°, 27.53° and 31.56° indicating a semi-crystalline morphology.^33,34^

**Fig. 4:**
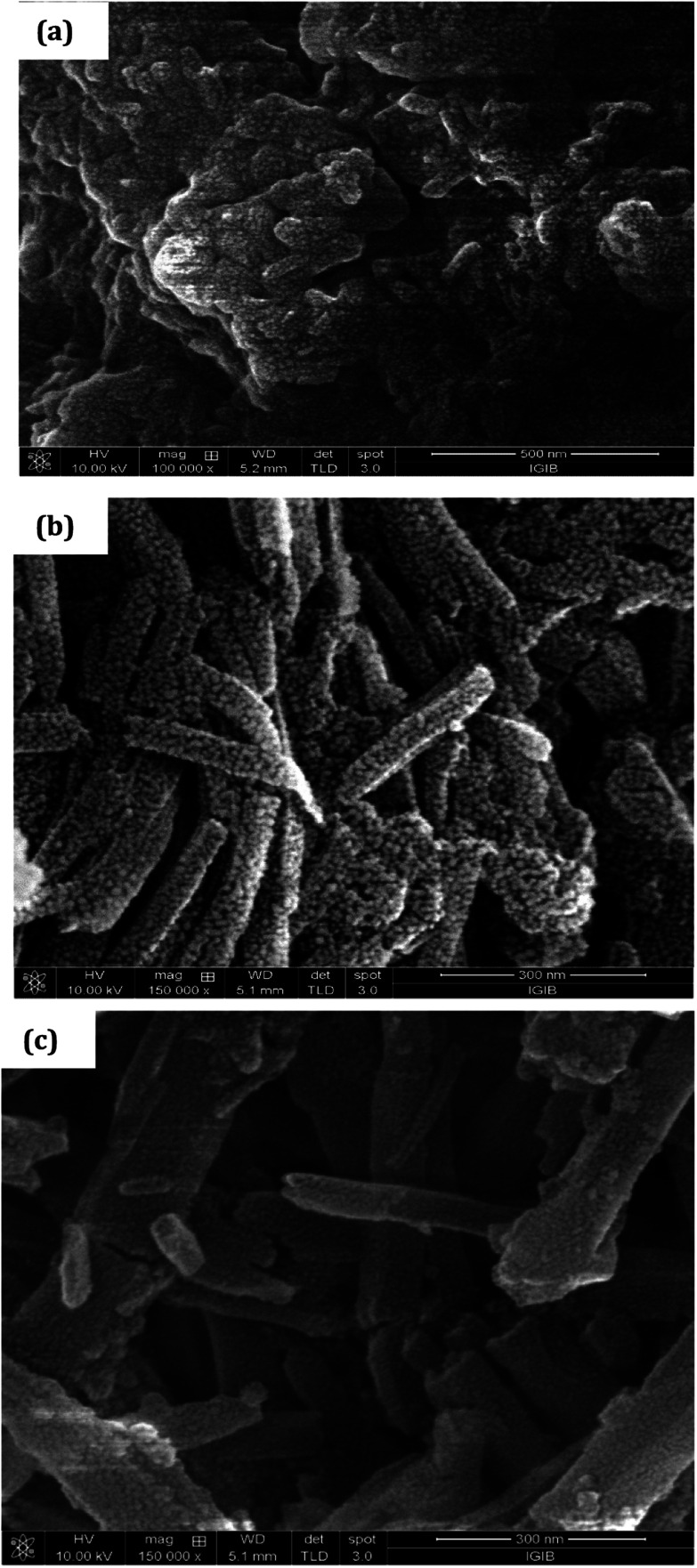
SEM of (a) PCR, (b) PCR-*co*-PANI, (c) PCR-*co*-POPD.

The surface morphology of PCR, Fig. 4(a), showed densely stacked granular aggregates that could be well correlated to its amorphous structure observed in the XRD pattern. The SEM of PCR-*co*-PANI, Fig. 4(b), showed the formation of fused rod like structures, while the SEM of PCR-*co*-POPD, Fig. 4(c), exhibited tubular hollow morphology. The rods were found to be non-uniformly stacked. It could be noticed that upon co-oligomerization, a self-assembled morphology was developed which was found to be dependent upon the co-monomer composition and was found to be in close agreement with the crystalline morphology depicted from XRD analysis.

### UV-visible and fluorescence characteristics of PCR and its co-oligomers

**Fig. 5:**
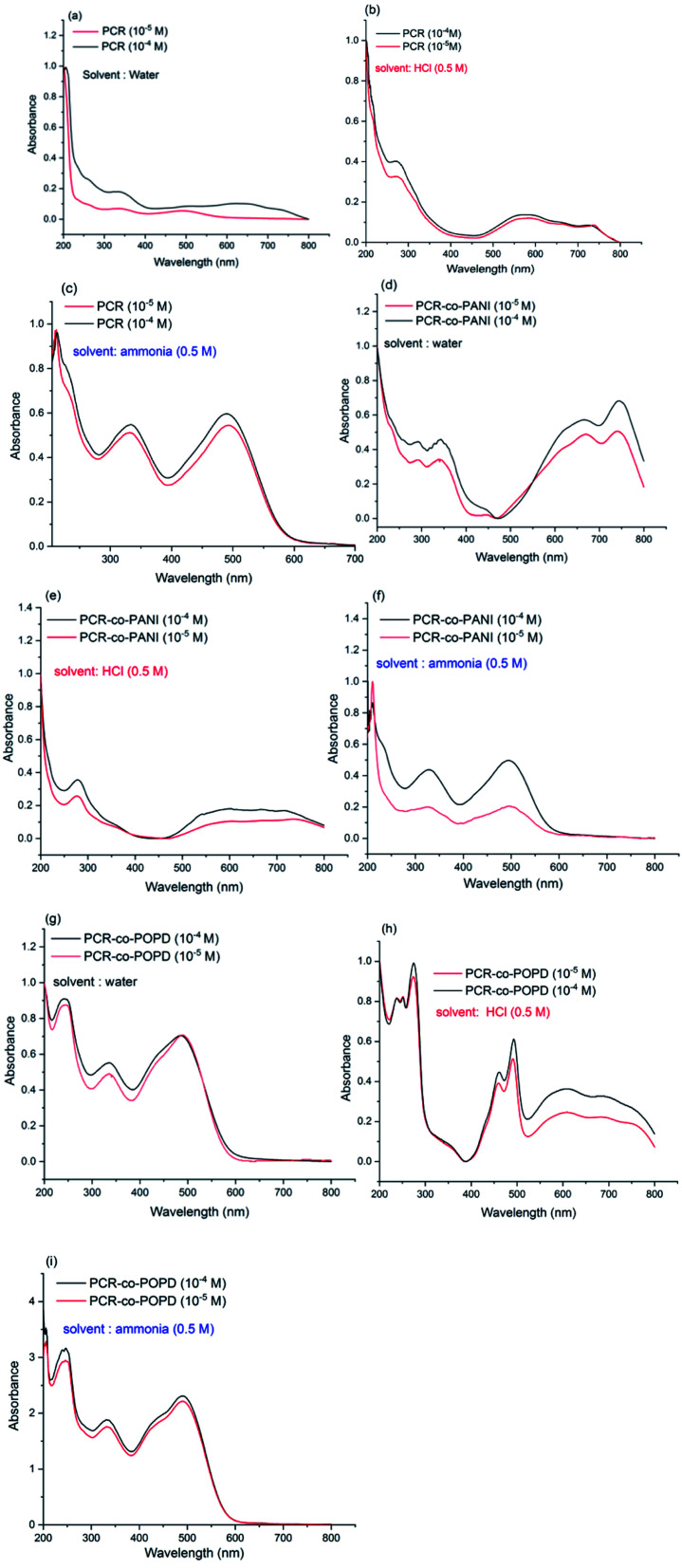
UV-visible spectra of (a) PCR (water medium), (b) PCR (acidic medium), (c) PCR (basic medium), (d) PCR-*co*-PANI (water medium), (e) PCR-*co*-PANI (acidic medium), (f) PCR-*co*-PANI (basic medium), (g) PCR-*co*-POPD (water medium), (h) PCR-*co*-POPD (acidic medium), (i) PCR-*co*-POPD (basic medium).

It is well-known that dyes undergo aggregation in solvent medium which highly depends on the pH of the solution. Hence, to investigate the effect of medium on the electronic transitions of the synthesized oligomers, the UV spectra were taken in water medium, basic medium (0.5 M ammonia) and acidic medium (0.5 M HCl). The spectrum of Congo red dye (shown in ESI as Fig. S3†) in water medium which revealed peaks at 210 nm, 340 nm and 500 nm. The later peak was associated with π–π* transition of azo group while the 340 nm was correlated to the π–π*-transition of –NH group. The changes in the absorption spectrum under basic and acidic conditions were attributed to self-aggregation tendency of the dye through π-stacking interactions over the benzene rings.^35^ The UV spectrum of PCR, PCR-*co*-PANI and PCR-*co*-POPD were recorded at two different concentrations of 10^−4^ M and 10^−5^ M in acidic, basic and neutral media and are shown Fig. 5(a–i). The UV spectrum of PCR (10^−4^M) in water revealed peaks at 350 nm, and a diffuse hump around 650 nm, while the spectrum of the oligomer taken at a concentration range of 10^−5^ M exhibited a small peak around 360 nm while the diffuse hump was noticed at 480 nm. The UV spectrum of the same oligomer taken in basic medium, Fig. 5(b), revealed peaks around 210 nm, 325 nm and 490 nm while the spectrum in acidic medium, Fig. 5(c), showed a diffuse peak around 270 nm and a broad hump centered at 580 nm. The theoretical spectrum was computed using water as solvent which revealed peaks at 360 nm and 455 nm (given in ESI as Fig. S4(a)†). As compared to CR dye, the peak in the visible region revealed a considerable shift in different media owing to self-aggregation. The common driving forces for the self-assembly of dyes are hydrophobic interactions, π-interactions, dispersion forces and H-bonding. The consequence of withdrawing electrons from polar substituents such as —sulfonic and —amino groups, increases the tendency to dissociate protons. The protonation of azo bond occurs at pH ranging between 7–8 while protonation of amino groups occurs at pH 4.5–5.5.^36^ The positive charge which appears as a result of protonation of the amino group balances the charge of sulfonic group and decreases the overall repulsion between molecules.

The UV-vis spectrum of PCR-*co*-PANI, Fig. 5(d), revealed peaks at 290 nm, 320 nm, 650 nm and 720 nm in neutral medium while the spectrum in acidic medium showed peaks at 280 nm and a tail extending up to 600 nm,Fig. 5(e). The transition associated with the peak at 720 nm could be correlated to the presence of PANI while the 600 nm peak was correlated to the π–π* transition of azo group. Protonated Congo red dye shows formation of two tautomers-ammonium form with the proton attached to the amino nitrogen and an azonium form, where the proton is added to the α-azo nitrogen. In the acidic solution, both forms are present in equilibrium mixture. The absorption band at 520 nm occurs when the ammonium form is dominant particularly in neutral and basic solutions while the azonium form appears when the formation of a quinoid structure takes place that causes absorption at higher wavelengths between 600–800 nm. Hence, the presence of long tail around 600 nm in acidic medium is associated with doping of PANI by PCR while in basic medium, Fig. 5(f), the PCR was noticed to exist in ammonium form and suppressed the peaks related to PANI.^37^ The theoretical spectrum of PCR-*co*-PANI (given in ESI as Fig. S4(b)†) showed peaks at 360 nm and 590 nm which were in close agreement with the peaks observed in water medium. The UV-vis spectrum of PCR-*co*-POPD, Fig. 5(g), revealed peaks around 240 nm, 330 nm, 490 nm and a broad hump centered at 600 nm in water medium, while in acidic medium, Fig. 5(h), peaks were noticed at 250 nm, 270 nm, 440 nm, 490 nm which were well correlated to the presence of PCR as well as POPD. The theoretical spectrum of PCR-*co*-POPD (given in ESI as Fig. S4(c)†) showed peaks at 350 nm and 500 nm which were in close agreement with the peaks observed in water medium. The UV-spectrum of PCR-*co*-POPD in basic medium, Fig. 5(i), exhibited peaks at 240 nm, 320 nm and 490 nm. The peak observed at 600 nm in water medium and 440 nm in acidic medium was associated with polaronic transitions of POPD as reported in previous studies and confirmed the co-oligomerization of POPD with PCR.^34,38^

The experimental oscillator strength values were calculated for PCR and its co-oligomers in all the media while theoretical oscillator strength values were computed only for oligomers in water medium as shown in Table 3. The oscillator strength values for PCR in neutral and acidic media were calculated to be 0.028 and 0.09 while the theoretically computed value was found to be 0.025. The oscillator strength values for PCR-*co*-PANI and PCR-*co*-POPD were observed to be highest in neutral medium which were in close agreement with the theoretically computed values. Expanded orientation of polymeric chains in water medium leads to addition of the net transition dipole moment causing increase in the oscillator strength values while in case of acidic/basic media, protonation/deprotonation of the dye as well as conducting polymer takes place which induces stress causing excessive entanglement of the chains and the net transition dipole moments will vectorially cancel out leading to lower oscillator strength values.

**Table 3:** Oscillator strength values of PCR, PCR-*co*-PANI and PCR-*co*-POPD

Samples (medium)	*λ*_max_ (nm) experimental (theoretical)	Exp. oscillator strength (theoretical oscillator strength)
PCR (water)	480 (455)	0.028 (0.025)
PCR (basic)	500	0.07
PCR (acidic)	660	0.09
PCR-*co*-PANI (water)	750 (590)	0.18 (0.17)
PCR-*co*-PANI (basic)	500	0.14
PCR-*co*-PANI (acidic)	660	0.12
PCR-*co*-POPD (water)	490 (500)	0.17 (0.16)
PCR-*co*-POPD (basic)	480	0.15
PCR-*co*-POPD (acidic)	660	0.13

**Fig. 6:**
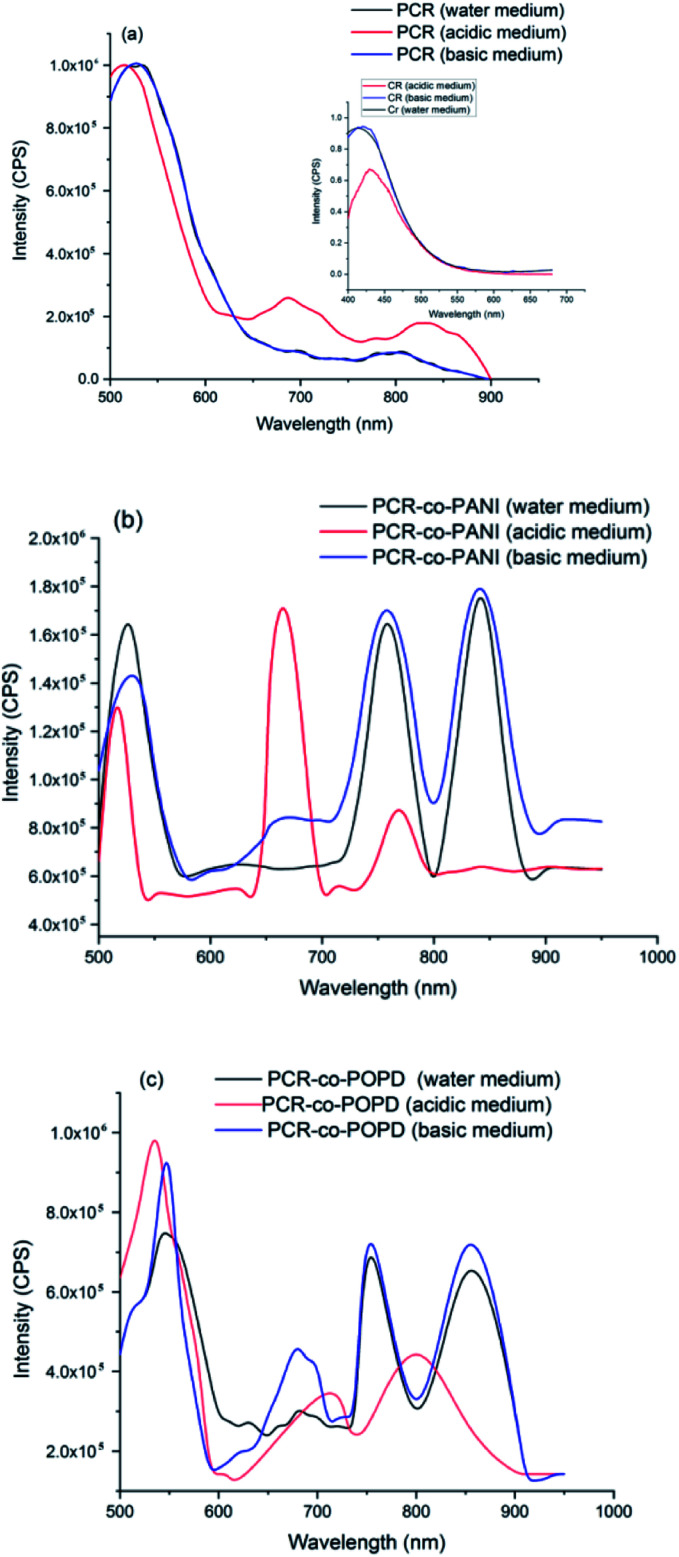
Fluorescence emission spectra of (a) PCR, (b) PCR-*co*-PANI and (c) PCR-*co*-POPD.

The emission spectra of PCR, PCR-*co*-PANI and PCR-co-POPD are shown in Fig. 6(a)–(c). Upon excitation at 480 nm, the emission spectrum of PCR in acidic medium revealed a prominent peak at 520 nm and broad peaks at 680 nm and 850 nm corresponding to S_1_ → S_0_ transition. The emission spectrum of pristine Congo red revealed emission at 450 nm upon excitation at 380 nm.

The differences in the emission peaks of PCR and pure dye could be attributed to increase in the extent of aggregation as observed in the UV-visible studies. The emission spectrum of PCR in basic and neutral media, Fig. 6(a), revealed peaks at 525 nm, a small peak around 700 nm and a broad hump at centered at 800 nm which was well correlated to the absorption spectra of the polymer.

The emission spectrum of PCR-*co*-PANI, Fig. 6(b), revealed a prominent peak 525 nm, 650 nm, 770 nm and 850 nm. The intensity as well as broadness was found to be higher in basic and neutral media as compared to acidic medium which showed peaks at 510 nm, 675 nm and 770 nm. The peaks observed around 510–520 nm were correlated to the presence of Cong Red unit while the emission peaks around 600 and 700 nm were associated with the presence of PANI. The shift in the emission peaks was attributed to the doping effect in acidic medium. Likewise, the emission spectrum of PCR-*co*-POPD, Fig. 6(c), revealed intense peaks at 550 nm, 670 nm, 760 nm and 850 nm in neutral and basic media while the peaks in acidic medium were observed around 520 nm, 710 nm and 805 nm respectively. It has been reported in literature that the emission spectrum of pure POPD exhibits peaks around 550 nm^3,33^ but in this case the peaks related to the POPD content in the co-oligomer were noticed at higher emission wavelengths due to co-oligomerization.

The quantum yield (*Φ*) values were calculated using rhodamine B as a reference, Table 4.^34^ The *Φ* values were found to be highest for PCR-*co*-POPD in basic medium, PCR in basic medium and PCR-*co*-PANI in acidic medium. Intense emission was observed in the region spanning between 800–900 nm which showed that the co-oligomers could tailored for designing NIR probes applicable in bioimaging.

**Table 4:** Fluorescence emission and quantum yield values of PCR, PCR-*co*-PANI and PCR-*co*-POPD

Polymer/co-oligomer	*λ*_max_ (nm)	Integrated area (*I*_sample_)	Quantum yield(*ø*)
PCR (neutral)	520	85 648 100	0.036 ±0.0 2
	800	69 521 400	0.028 ±0.0 2
PCR (acidic)	515	65 049 000	0.025 ± 0.0 1
	831	14 803 200	0.014 ± 0.02
PCR (basic)	525	75 622 200	0.037 ± 0.01
	850	76 153 300	0.034 ± 0.02
PCR-*co*-PANI (neutral)	525	29 193 700	0.024 ± 0.02
	850	96 099 100	0.045 ± 0.03
PCR-*co*-PANI (acidic)	525	84 806 400	0.035 ± 0.02
	770	84 732 200	0.034± 0.03
PCR-*co*-PANI (basic)	525	16 387 800	0.015± 0.03
	850	38 253 000	0.028 ± 0.02
PCR-*co*-POPD (neutral)	550	44 390 500	0.022± 0.02
	850	35 459 000	0.019± 0.02
PCR-*co*-POPD (acidic)	520	65 489 700	0.028± 0.02
	805	49 693 600	0.024± 0.02
PCR-*co*-POPD (basic)	550	86 358 400	0.039± 0.02
	850	59 055 200	0.023± 0.02

### Analysis of cytotoxicity and imaging of tumor cells

**Fig. 7:**
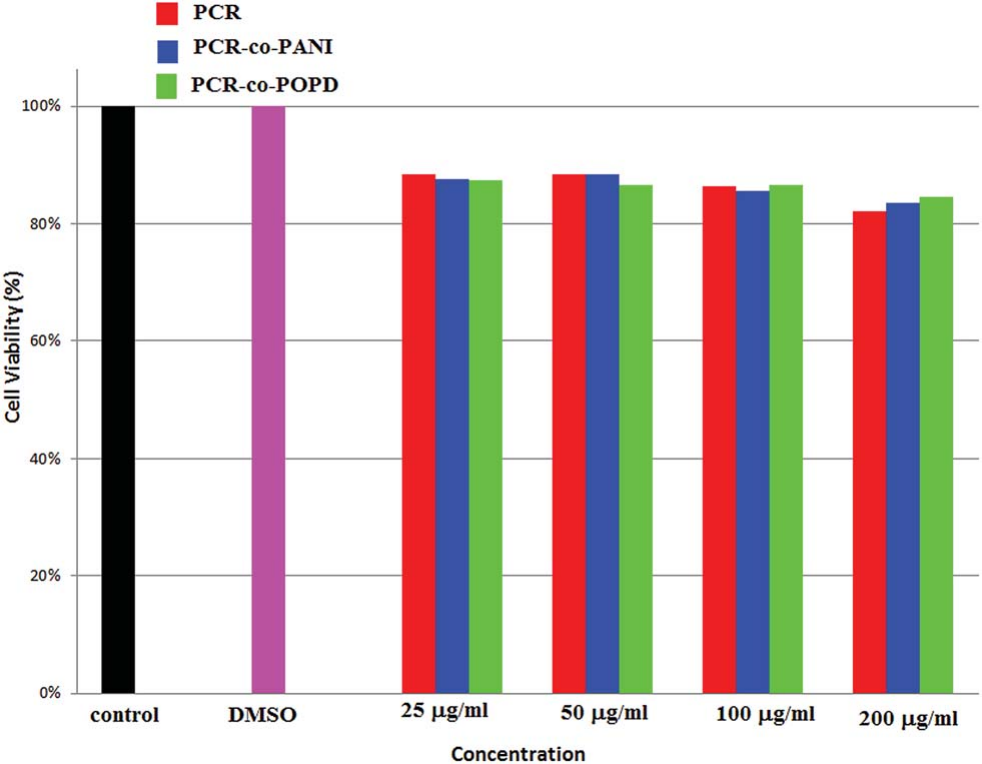
Percent cell viability profile of PCR and its co-oligomers.

Human cervical cancer (HELa) tumor cells as well as normal cells were used to assess the cytotoxicity. The effects of homopolymer and co-oligomers on cell viability were measured using the MTT assay. The homopolymer and co-oligomers of PCR showed insignificant toxicity up to concentration range of 250 μg mL^−1^, Fig. 7. The tumor cell lines clearly showed the capability to maintain membrane integrity upon exposure to high dosages of co-oligomers and could therefore be safely used at concentrations as high as 250 μg mL^−1^.

**Fig. 8:**
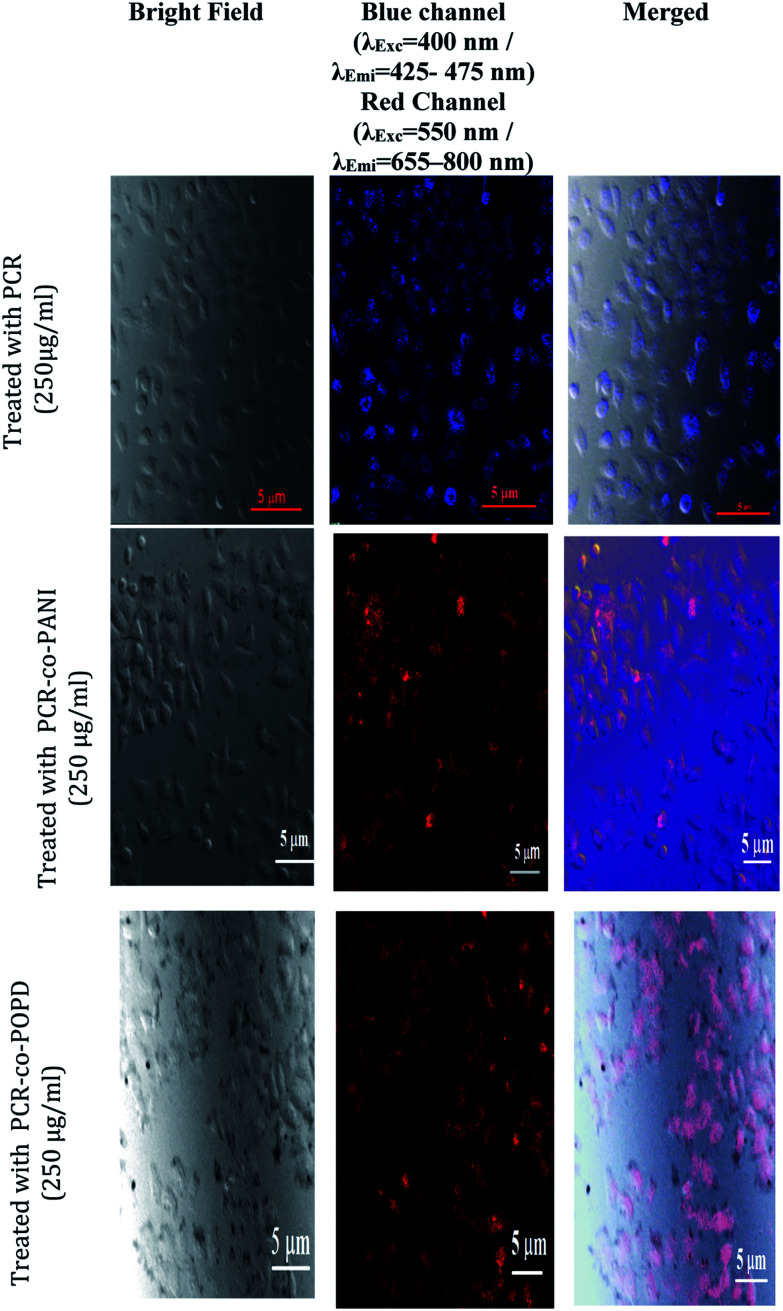
Fluorescence imaging of HeLa cells incubated with PCR, PCR-co-PANI and PCR-co-POPD co-oligomers for 24 h.

The PCR, PCR-*co*-PANI and PCR-*co*-POPD treated HeLa cells were visualized *via* confocal microscopic imaging to explore their staining ability as well as their capability to detect tumor cells, Fig. 8. Blue luminescence was observed for HeLa cells treated with PCR after 24 h while the co-oligomers of PCR exhibited red emission. The entire cell was uniformly stained which confirmed the amphiphilic nature of the oligomers to spontaneously insert and fuse into intracellular biological membranes. It can therefore be concluded that the oligomers were successfully internalized and dissociated to emit blue and red fluorescence. The live cell imaging could be tuned with blue, red or even green fluorescence by varying the structure of polymers. The fluorescence signals were noticed to be intense from the nucleus and cytoplasm of the HeLa cells even after 24 h treatment with PCR-*co*-PANI and PCR-*co*-POPD.

## Conclusion

Congo red based oligomer and co-oligomers were successfully synthesized *via* chemical polymerization. The ^1^H-NMR and IR studies confirmed the polymerization of Congo red with aniline and *o*-phenylenediamine. The theoretical results were found to be in close agreement with the experimentally observed data. XRD results confirmed semi-crystallinity while SEM exhibited self-assembled morphology forming tubular rod like structures. The co-oligomers PCR-*co*-PANI and PCR-*co*-POPD revealed fluorescence emission around 700–800 nm upon co-oligomerization with Congo red. The cell viability testes revealed that the co-oligomers could be safely used up to 250 μg mL^−1^. Live cell imaging analysis showed intense red emission from the HeLa cells when treated with PCR-*co*-PANI and PCR-*co*-POPD. The co-oligomers could therefore be used as effective bioimaging agents. Studies on the photodynamic activity of these NIR co-oligomers are underway in our laboratory and will be published soon.

## Conflicts of interest

There are no conflicts to declare.

## Supplementary Material

RA-009-C9RA05814A-s001
