# Supplementary material for: Development of a near infrared novel bioimaging agent via co-oligomerization of Congo red with aniline and o-phenylenediamine: experimental and theoretical studies
Source: RSC Adv. 2019 Nov 13;9(62):36479–91. doi: 10.1039/c9ra05814a (PMC9075138; doi:10.1039/c9ra05814a)
Supplement: RA-009-C9RA05814A-s001 [file RA-009-C9RA05814A-s001.pdf]

Electronic Supplementary Material (ESI) for RSC Advances.
This journal is © The Royal Society of Chemistry 2019

Supporting information

Development of Near Infrared Novel Bioimaging Agent via Co-oligomerization of Congo Red with Aniline and *o*-phenylenediamine: Experimental and Theoretical Studies

Neetika Singh[a], Prabhat Kumar[b] Raj Kumar[c] ,Elham A. Aazam[d] and Ufana Riaz*[a]

[a] Materials Research Laboratory Department of Chemistry, Jamia Millia Islamia, New Delhi-110025, India, [b] Advanced Instrumentation Research Facility, Jawaharlal Nehru University, New Delhi- 110067, [c] School of Life Sciences, Jawaharlal Nehru University, New Delhi- 110067,[d] Chemistry Department, Faculty of Science, King Abdul Aziz University, Jeddah 23622, Saudia Arabia

*Corresponding author email: ufana2002@yahoo.co.in

Table S1 Intrinsic Viscosity and average molar mass of PCR, PCR-co-PANI, and PCR-co-POPD

Homopolymer/copolymers	Intrinsic viscosity (η)	Viscosity average molar mass (Mv)
PCR	0.11	5117 ± 3
PCR-co-PANI	0.13	6179 ± 2
PCR-co-POPD	0.15	6814 ± 2

(a)

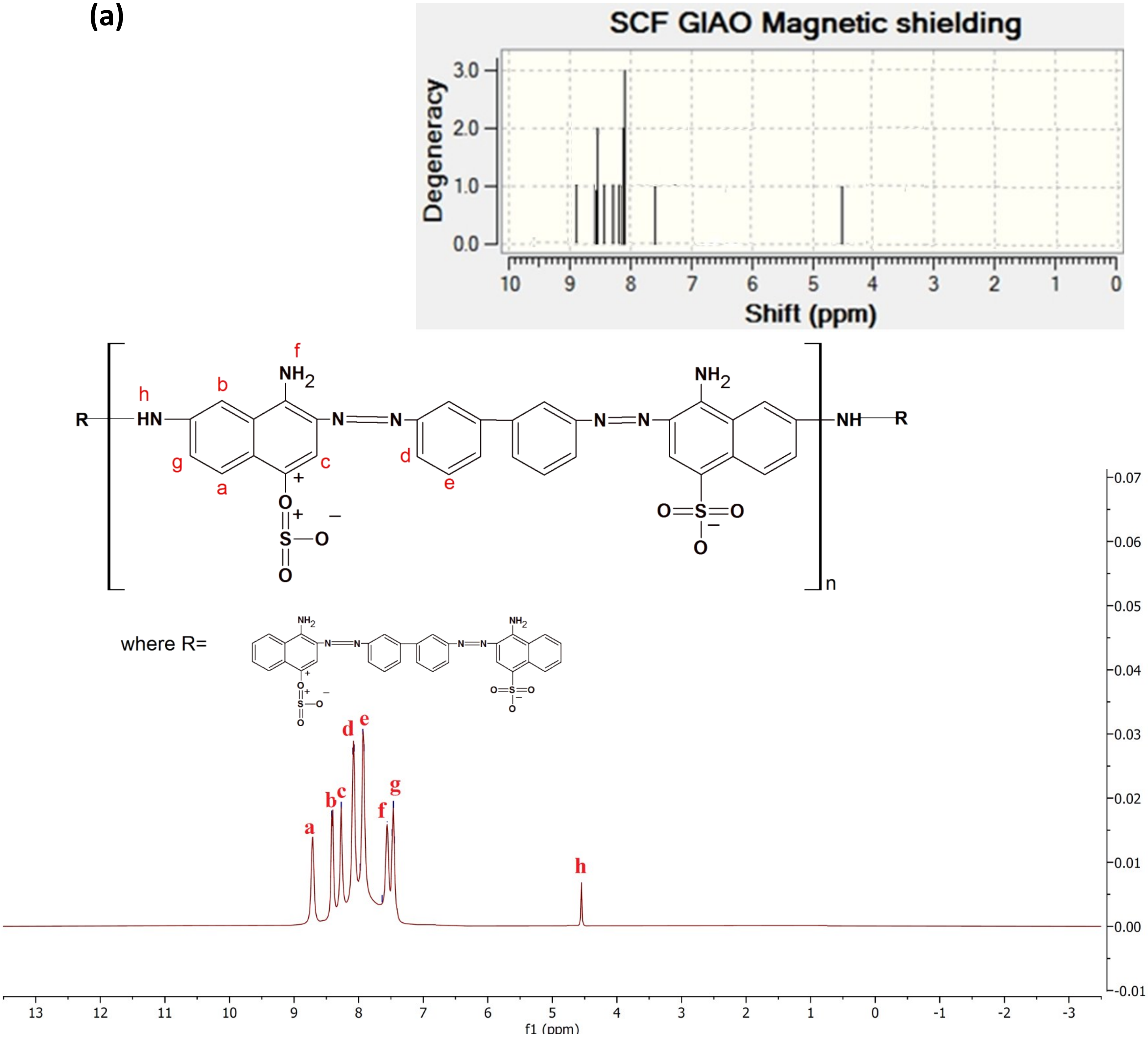

SCF GIAO Magnetic shielding
Degeneracy
Shift (ppm)
where R=
a
b
c
d
e
f
g
h
n
f1 (ppm)

(b)

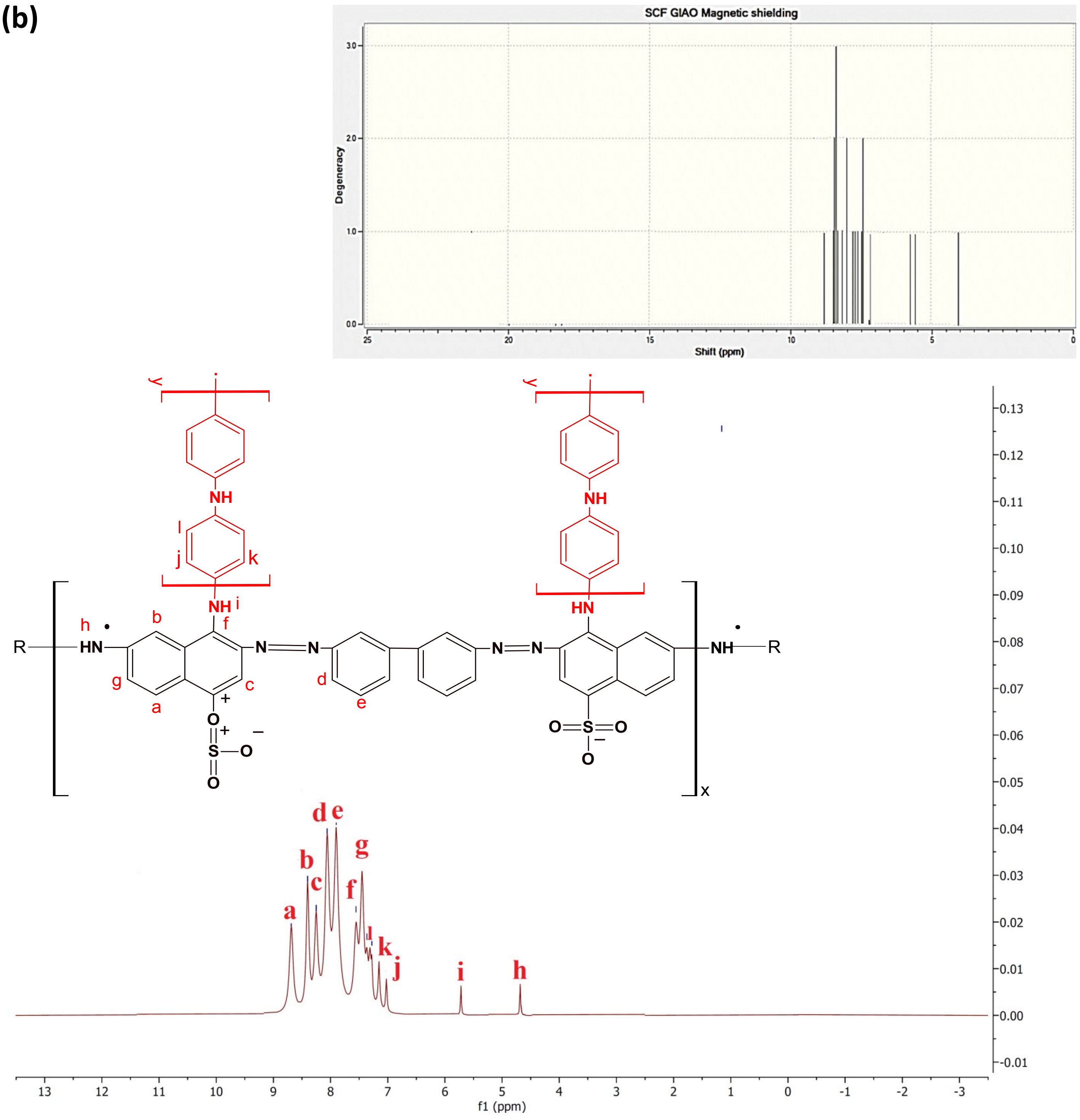
SCF GIAO Magnetic shielding
Degeneracy
Shift (ppm)
R
HN
NH
HN
NH
N=N
O=S=O
y
x
a b c d e f g h i j k l
f1 (ppm)

(c)

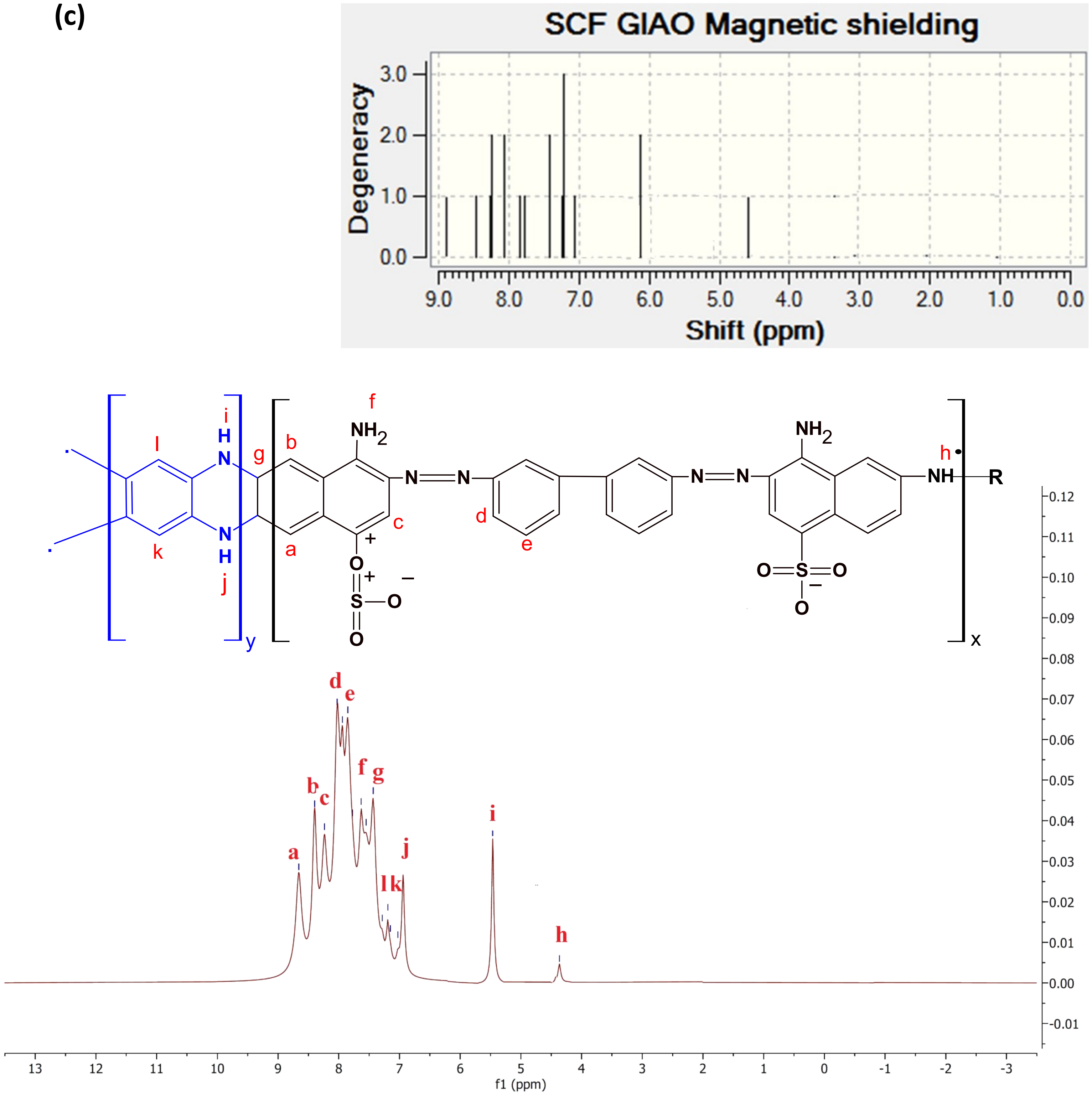

Figure S1 ^{1}H-NMR spectra of (a) PCR (b) PCR-co-PANI, (c) PCR-co-POPD

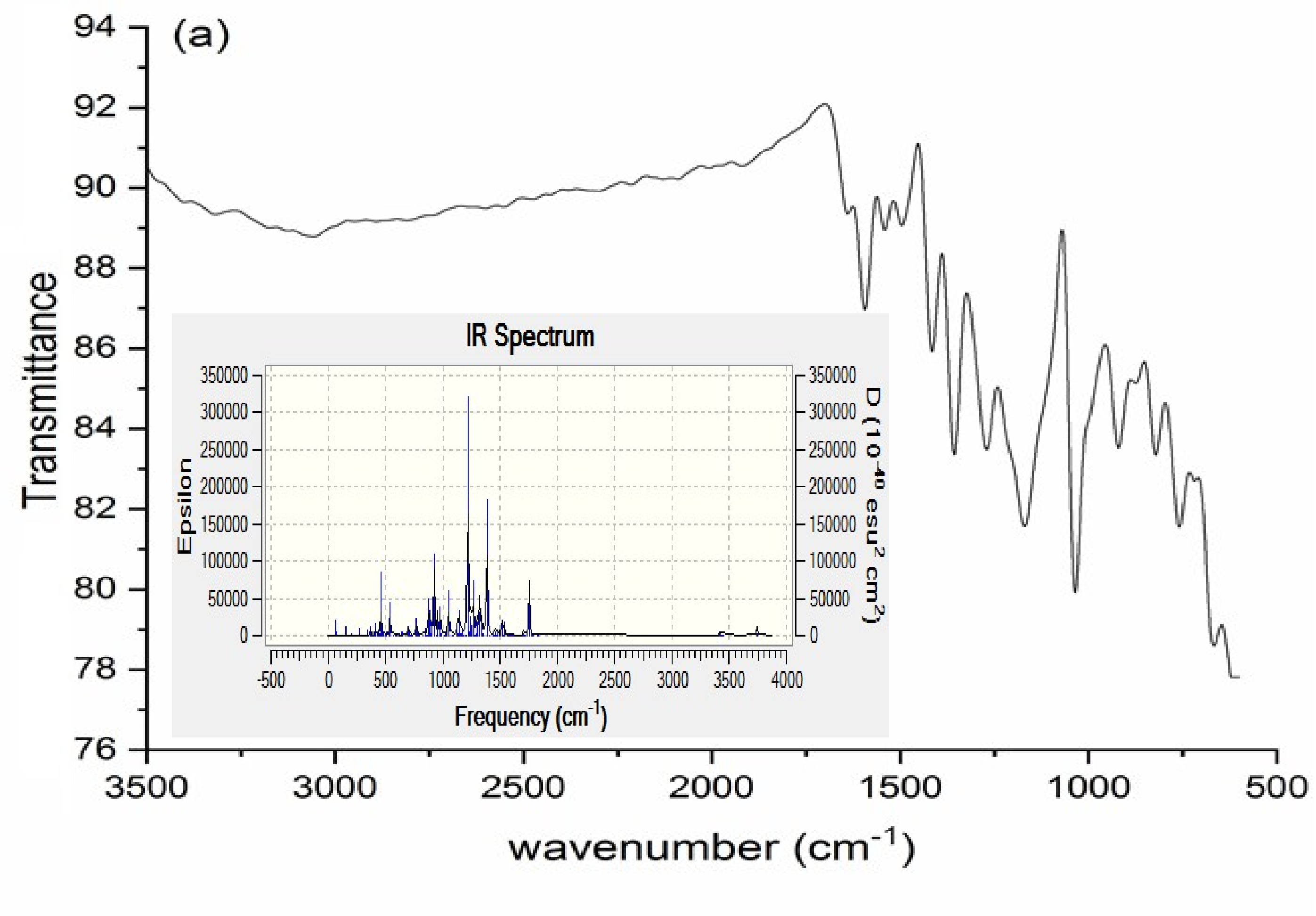
(a)
Transmittance
wavenumber (cm-1)
94
92
90
88
86
84
82
80
78
76
3500
3000
2500
2000
1500
1000
500
IR Spectrum
Epsilon
D (10-40 esu2 cm2)
Frequency (cm-1)
350000
300000
250000
200000
150000
100000
50000
0
-500
0
500
1000
1500
2000
2500
3000
3500
4000

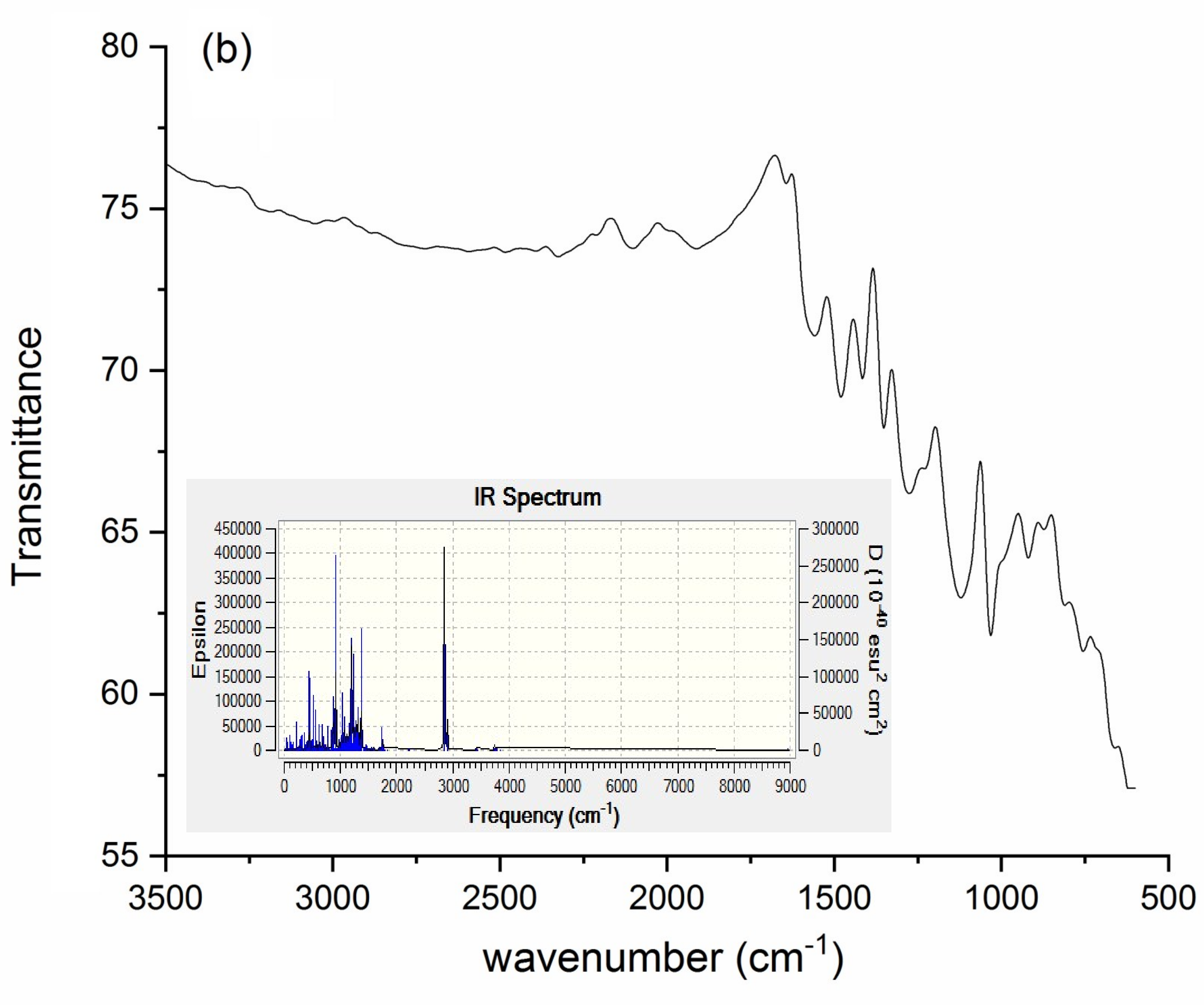

(b)
Transmittance
wavenumber (cm-1)
80
75
70
65
60
55
3500
3000
2500
2000
1500
1000
500
IR Spectrum
Epsilon
D (10-40 esu2 cm2)
Frequency (cm-1)
450000
400000
350000
300000
250000
200000
150000
100000
50000
0
300000
250000
200000
150000
100000
50000
0
1000
2000
3000
4000
5000
6000
7000
8000
9000

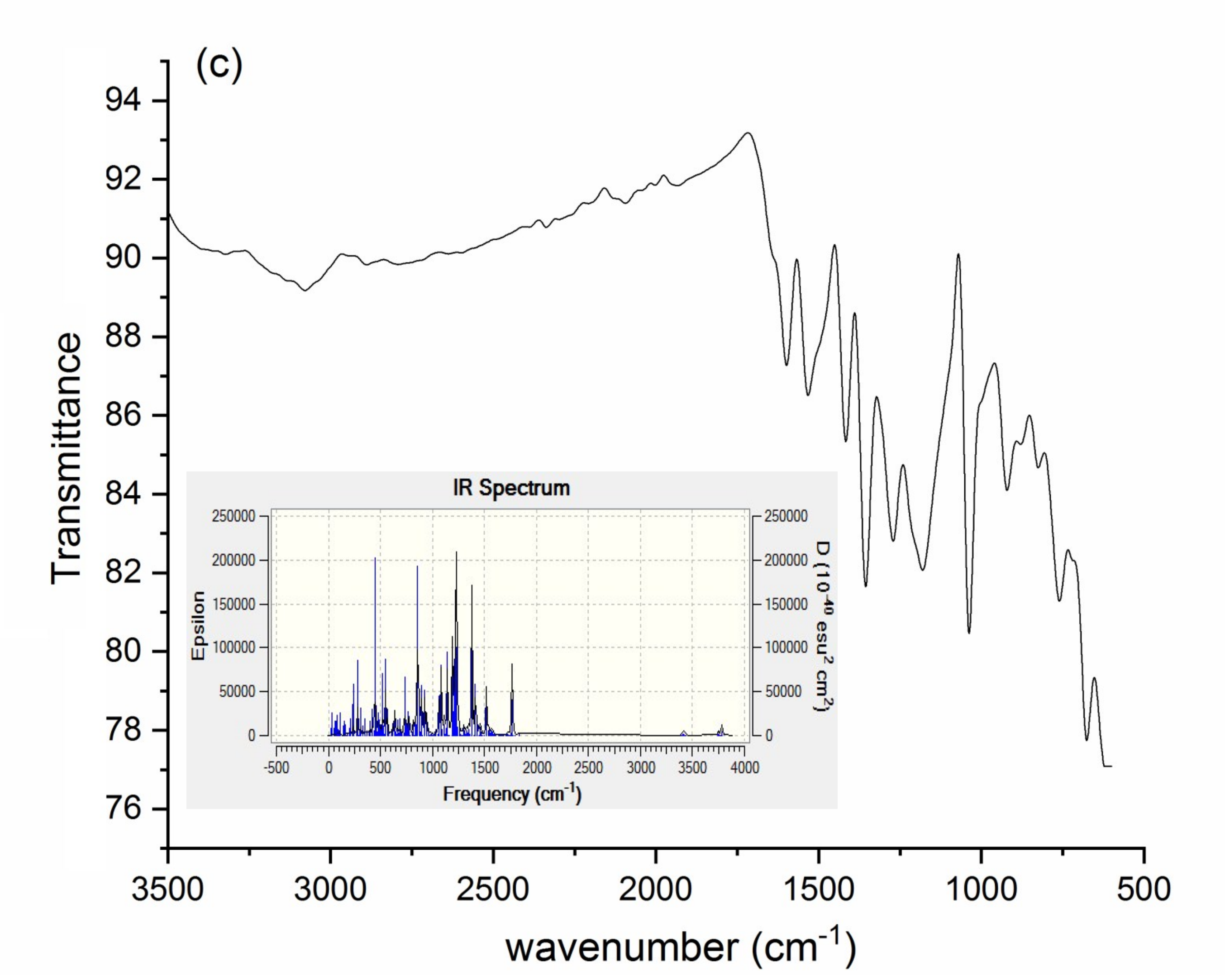

Figure S2 IR spectra of (a) PCR (b) PCR-co-PANI, (c) PCR-co-POPD

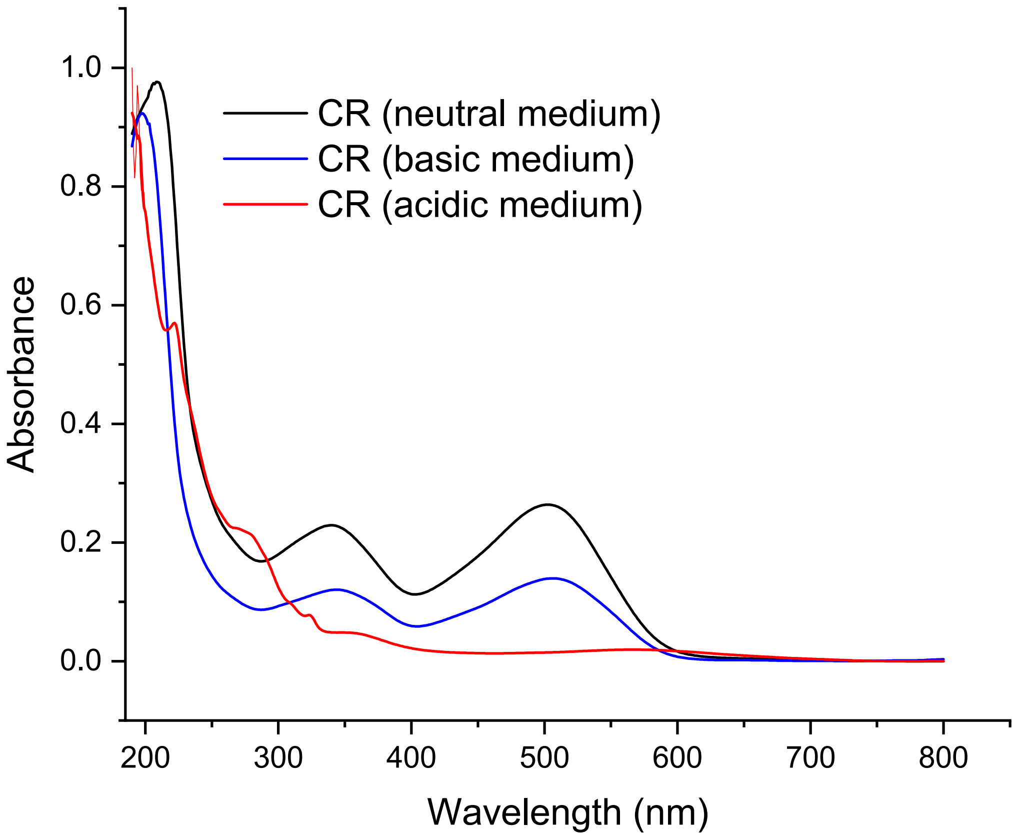

Figure S3 UV –visible spectra of Congo Red in acidic, basic and neutral media

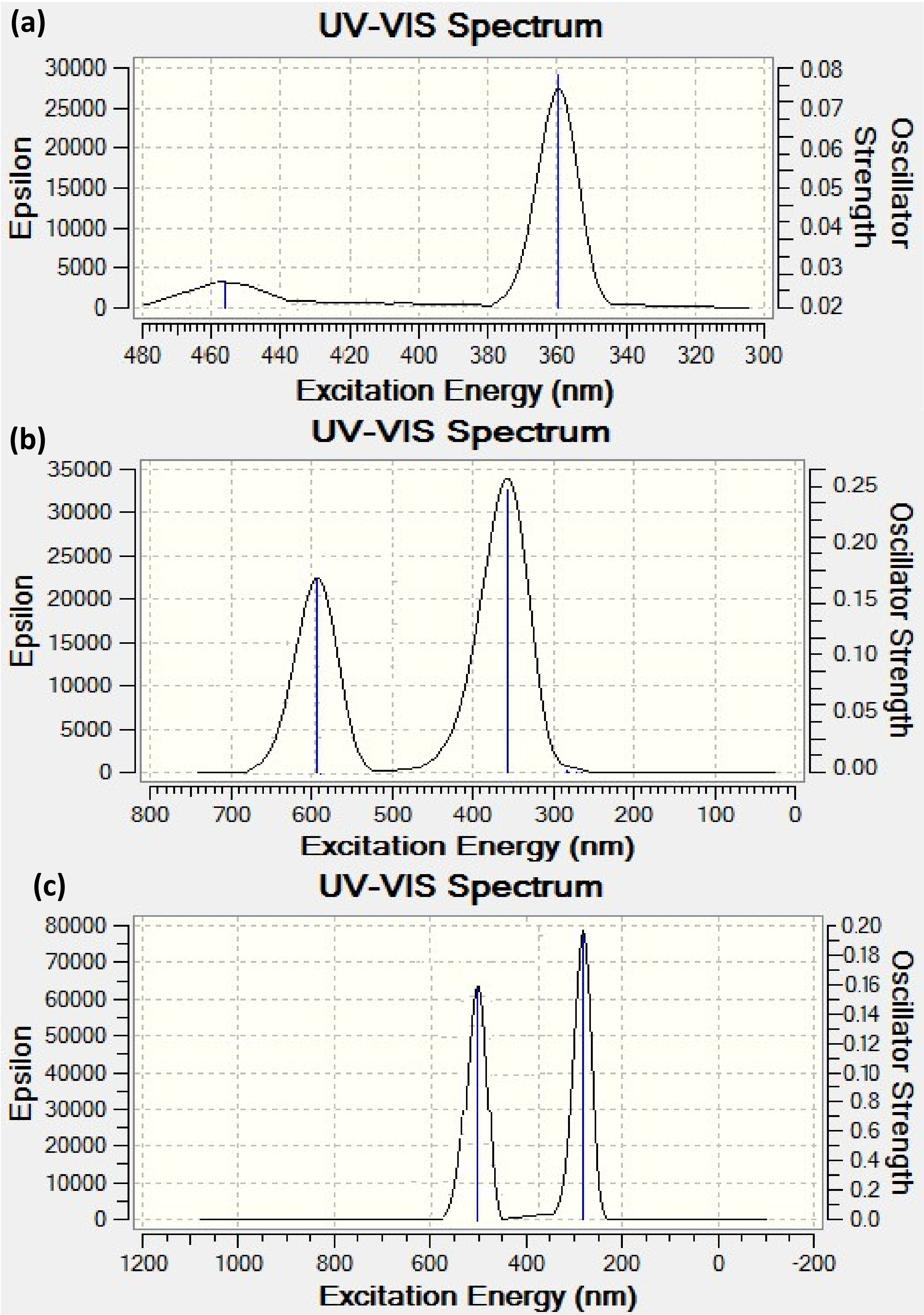

Figure S4 Theoretical UV –visible spectra of (a) PCR,(b) PCR-co-PANI, (c) PCR-co-POPD
